# Facial Diplegia—Complication or Manifestation of SARS-CoV-2 Infection? A Case Report and Systemic Literature Review

**DOI:** 10.3390/healthcare9111492

**Published:** 2021-11-02

**Authors:** Anna K. Szewczyk, Urszula Skrobas, Anna Jamroz-Wiśniewska, Krystyna Mitosek-Szewczyk, Konrad Rejdak

**Affiliations:** 1 Department of Neurology, Medical University of Lublin, ul. Jaczewskiego 8, 20-954 Lublin, Poland; skrobas.urszula@gmail.com (U.S.); annajamrozwisniewska@umlub.pl (A.J.-W.); krejdak@yahoo.com (K.R.); 2 Department of Child Neurology, Medical University of Lublin, ul. Profesora Antoniego Gębali 6, 20-093 Lublin, Poland; krystyna.mitosek@gmail.com

**Keywords:** COVID-19, SARS-CoV-2, Guillain-Barré Syndrome, peripheral nervous system disease, peripheral facial nerve palsy, Bell’s palsy, COVID-19 vaccination

## Abstract

Since the outbreak of the new coronavirus, healthcare systems around the world have witnessed not only COVID-19 symptoms but also long-term complications of the aforementioned, including neurological problems. We report a clinical case of an adult patient with bilateral facial nerve palsy and progressive ascending paresis of the limbs after contracting the novel coronavirus (COVID-19). Additionally, the systematic review aimed to identify and summarize specific clinical features, outcomes and complications of the studies focusing on bilateral facial diplegia as a sequela of COVID-19 infection. The total number of analyzed patients was 15. Only one patient was diagnosed with isolated bilateral palsy; the rest had Guillain-Barré Syndrome (GBS). With one exception, all the presented cases had favorable outcomes, with facial palsy recovery from slight to almost complete. In patients with a confirmed COVID-19 diagnosis, bilateral facial palsy may be an isolated symptom as well as a variant of GBS. Symptoms of cranial nerve damage during a COVID-19 infection may explain the appearance of facial nerve damage. In order to clarify the spectrum of neurological manifestations and a causal relation between SARS-CoV-2, COVID-19 vaccination and neurological symptoms, direct attention towards the study of this virus is crucial. It seems reasonable to recognize human coronavirus as another potential GBS trigger.

## 1. Introduction

Since the outbreak of the new coronavirus, healthcare systems around the world have witnessed not only COVID-19 symptoms but also long-term complications of the aforementioned. New phenomenon might be overviewed or misdiagnosed due to its novelty or lack of knowledge about the connection to previous severe acute respiratory syndrome coronavirus 2 (SARS-CoV-2) infection, which might have serious consequences including death. There is a need to share knowledge and increase awareness among health care professionals for better management of the patients. 

The first reported patient with SARS-CoV-2 in Wuhan, China was reported in December 2019 [1]. The coronavirus family is a group of single-stranded RNA viruses causing respiratory and intestinal infections in the host [2]. In humans, asymptomatic to severe pneumonia leading to death can be observed, as well as fever, cough and myalgia. However, atypical presentation of the disease is not uncommon [3].

Neurological manifestation related to SARS-CoV-2 and other coronaviruses depends on the exact location of the lesion, and their symptoms range from mild to severe. Among peripheral nervous system (PNS) alterations, neuralgia, olfactory and gustatory disorders are mentioned. Assessing the changes in the central nervous system (CNS), symptoms such as headache, dizziness, altered consciousness and ischemic or hemorrhagic stroke were observed. Acute inflammation of the CNS (brain, spinal cord and meninges) and PNS were also repeatedly reported [4,5]. 

## 2. Case Report

A 70-year-old Caucasian male, a proper diction teacher with a medical history of hypertension and stable heart disease, was admitted to our neurological department in November 2020 with acute symmetric progressive ascending paresis of the limbs, impaired sensation and bilateral facial nerve palsy. One month earlier (on 20 October 2020) he was treated at home for suspected SARS-CoV-2 infection without serious complications. The infection manifested itself through fever, gastrointestinal symptoms, headaches and severe pain in the thoracic spine. Three weeks later, the patient had an antibody blood test for SARS-CoV-2 with immunoglobulin M (IgM) rate at 4.18 (negative result < 1.00) and immunoglobulin G (IgG) rate at 5.69 (negative result < 1.4). Seven days after the disease laboratory confirmation, he came to the GP because of paraesthesia of the distal parts of the limbs and burning of the tongue. A diagnosis of ulcers (aphthae) in the mouth and sensory disturbances due to changes in the cervical and lumbar spine were made. Then, two days later, bilateral paresis of the facial nerve appeared, and he was thus transported from the private neurological praxis by ambulance to our neurology department.

The systemic examination was normal. In the neurological examination the patient presented the following: complete lower motor neuron facial weakness bilaterally, swallowing difficulties, jaw dropping and dysarthria, symmetrical flaccid quadriparesis, absent deep tendon reflexes and sensory disturbances from the thoracic spinal nerve 6 (T6) downwards. The patient complained of a lack of facial expressions and diplopia. Then, due to diaphragm and intercostal muscles paralysis, respiratory disorders of the “compression of the iron rim” type appeared. Brain computed tomography (CT) and magnetic resonance imaging (MRI) were performed without any acute pathological finding. In a thoracic MRI (scope of the study T6–L2) examination, due to striped, subtle contrast enhancement at T12–L1 level on the anterior and dorsal surface of the conus medullaris and the roots of the cauda equina, suspicion arose of Guillain-Barré Syndrome. A lumbar puncture was performed, and the CSF picture revealed normal glucose and cell count with 98 mg/dL protein level, albumin level at 56.8 mg/dL and positive oligoclonal bands type IV indicative of damage to the blood–brain barrier. Other laboratory tests were within normal range. A nerve conduction study showed sensorimotor polyneuropathy of a demyelinating-axonal character. The patient received intravenous treatment with immunoglobulins at a dose of 2 g per kilogram body weight; the course of treatment was uneventful and there was gradual improvement. For further recovery, the patient was transferred to the rehabilitation department.

## 3. Materials and Methods

We report a clinical case of patient with acute symmetric progressive ascending paresis of the limbs and bilateral facial nerve palsy after COVID-19 infection. The primary outcome of literature analysis was to identify and summarize specific clinical features and outcomes in SARS-CoV-2 patients complicated by bilateral facial diplegia. Our review focuses on the disease entities that lead to the emergence of bilateral facial diplegia, their likely underestimation and the impact of SARS-CoV-2 infection and COVID-19 vaccinations on their appearance. To identify appropriate literature, a systematic literature search was conducted based on the PRISMA guidelines [6]. The electronic databases PubMed and Google Scholar were searched for all adult (≥18 years) clinical case or case series descriptions or original articles about COVID-19 associated with bilateral facial nerve palsy, published before 30 June 2021. The studies were published in English, French or Polish. The following keywords or MeSH including all commonly used abbreviations of these terms were used: “bilateral facial nerve palsy”, “facial diplegia”, “bilateral facial nerve palsy”, “COVID-19”, “SARS-CoV-2”, “Guillain-Barré Syndrome”, “acute inflammatory demyelinating polyneuropathy”. For this analysis, we included only clinical studies containing neurological manifestations associated with a COVID-19/SARS-CoV-2 infection. Studies concerning other human coronaviruses or duplicating the cases already mentioned, as well as studies with unconfirmed COVID-19 cases and unilateral facial palsy, were excluded. The search, based on the titles and abstracts of all reports identified through electronic databases, was conducted by two reviewers independently to identify the studies matching the assumed criteria. In case of uncertainty, the full text of the article was obtained and discussed. At the time of writing, we found 14 cases of bilateral facial nerve palsy combined with SARS-CoV-2 infection, which are summarized in Table 1. Two reports for which only abstracts were available were nevertheless included due to meeting the criteria. The results of the statistical analysis are expressed as mean ± standard deviation. The authors found no review reports about the relationship between bilateral facial palsy and COVID-19 infection.

## 4. Results

This study reported a patient with progressive neurological manifestations in the form of ascending paresis of the limbs and impaired sensation that were initially misdiagnosed. The onset of the neurological symptoms appeared one month after COVID-19 infection, confirmed by the presence of IgG to SARS-CoV-2 in the patient’s blood. It was found that 100% patients reached positive virus-specific IgG after approximately 17–19 days after first symptoms [7]. 

Fourteen clinical cases were included in this systemic review, with a total of 15 analyzed patients, including the above clinical case. The age of the patients ranged from 20 to 70 years old, the average age was 46.13 ± 14.61 and the group was predominantly male (66.7%). The time elapsed between first symptoms of infection and admission to hospital due to neurological signs ranged from 2 days to 6 weeks with an average of 18.36 ± 10.82 days. Most cases (9/14) had a time lapse between 10 and 21 days. Of the available data, three patients had no symptoms of COVID-19 infection prior to admission, while four patients had a positive RT-PCR SARS-CoV-2 test result. Out of 15 described patients, only one had no albumin-cytological dissociation (ACD) in CSF results and was diagnosed with isolated bilateral facial palsy (BFP) in the context of otherwise asymptomatic COVID-19. Among the other patients, four presented only bilateral facial paresis with no other deviations in the neurological examination. In 5 of the 15 cases, brain MRI showed bilateral contrast enhancement of the facial nerves. Neurophysiological studies results were available for 10 out of 15 patients. The most common finding was demyelinating polyneuropathy. In the treatment, the following were used: intravenous immunoglobulins (IVIG) in 60.00% (9/15) of patients, corticosteroids in 33.33% (5/15) and plasmapheresis in 6.67% (one case). Data are missing in one case. Fourteen out of fifteen cases reported an improvement from slight to almost complete; however, one patient died from severe autonomic dysfunction (Table 1).

## 5. Discussion

The first clinical case of a patient with GBS and SARS-CoV-2 infection was presented in The Lancet Neurology Journal [20]. On 23rd January 2020, a 61 year-old woman, who had returned from Wuhan 4 days earlier, presented fatigue and rapidly progressive weakness in both legs, with no different general symptoms. Over time, the symptoms advanced to areflexia, with sensory disturbances and upper limb involvement. Lymphocytopenia and thrombocytopenia in the blood test and CSF albumin-cytological dissociation were reported in laboratory results. The results of nerve conduction studies supported diagnosis of demyelinating neuropathy and GBS. Treatment with immunoglobulins was applied; nevertheless a few days later, due to infectious symptoms, she tested positive for COVID-19 via RT-PCR testing and was transferred to an isolation room. 

Guillain-Barré Syndrome, despite being a rare disease, is diagnosed at any age and is the most common cause of acute flaccid paresis [21]. GBS incidence varies considerably with geographic location, with ranges between 0.4 and 3.25 or 0.6 to 4.0 cases per 100,000 people [22,23]. In children, the incidence rate is lower, approx. 0.69 per 100,000 person per year [24]. Referring to research conducted in Europe, a population-based study pointed out an incidence rate of 1.2–1.9 per 100,000 people [25]. This disease affects slightly more men than women and appears at around 40 years of age. The underlying cause of the higher incidence of the disease in men is not yet known [26,27]; however, this predominance seems to increase with age [28]. A higher proportion of the pure motor type is observed in younger patients, whilst the sensorimotor type increases with age [29]. Seasonal changes are also described, providing a higher incidence rate in winter compared to in summer [30,31]. Perhaps this seasonal variability can be linked with several demographic, immunological or environmental factors, including climatic conditions conducive to seasonal infections, like gastrointestinal and respiratory tract infections [32].

The relationship between COVID-19 and GBS and the resulting symptoms continue to be investigated. The vast majority of studies were published as case reports or series of cases [33,34,35]. Abu-Rumeileh et al. presented the clinical cases of 73 patients with confirmed COVID-19 history, in which he referred to 52 articles; Sriwastava et al. identified 50 GBS cases from 37 studies, while Finsterer et al. reviewed at least 220 patients from 95 papers at the end of December 2020 [36]. The number of patients experiencing GBS in the context of COVID-19 appears higher than anticipated, though there are still no unequivocal results. The similarity of the demographic results obtained in this review may support SARS-CoV-2 having a role in triggering GBS.

On the one hand, the number of registered GBS cases, while underestimated, has fallen during the COVID-19 pandemic; on the other hand, altered social conduct is observed. Due to changes in social behavior, such as social distancing and the use of personal protective equipment and hygienic procedures, the prevention of infectious diseases, known as triggers of GBS, has increased [37,38]. Recent comments characterizing GBS as occurring in occasional clusters can be found as well [39]. Another study presented results with neither an observed raise in the incidence of GBS in pandemic nor epidemiological evidence that SARS-CoV-2 is a causative factor for GBS [40]. The limited access to the healthcare and risk of co-infection in COVID-19 patients may also distort statistics [37]. Therefore, the exact number of GBS cases in the pandemic is difficult to estimate and long-term observation is still required.

Multiple potential “classical” triggers of GBS have been reported e.g., *Campylobacter jejuni* (C. jejuni), cytomegalovirus, Epstein–Barr virus, influenza and Zika virus infection, or vaccination. After a bacterial or viral infection, a cross-reaction called molecular mimicry appears, in which antibodies and nerve ending antigens are involved [41,42]. As one of the best-known virulence factors, sialylation of lipo-oligosaccharides (LOS) of the Gram-negative bacterium *Campylobacter jejuni* is mentioned. Its molecular similarity to ganglioside structures (GM1) on human spinal nerve roots drives immune-mediated nerve damage. As many as one-fourth or one-third of patients after this infection can develop GBS [43]. There are several classes of LOS; however, three of them—A, B and C—are isolated from GBS-patient stool. The first is associated with GBS and the second with Miller Fisher syndrome (MFS) [44]. 

In COVID-19 and neurological damage, three pathogenic pathways are proposed: direct damage, dysregulated inflammatory response and antibody-mediated injury. Freire et al. suggest neuro-invasive ability by disrupting the blood–brain barrier (BBB). Pro-inflammatory cytokines increase BBB permeability and activate glial cells [45] or retrograde axonal transport through the olfactory nerve or the enteric nervous system. Elevated neuroinflammatory parameters in serum and/or CSF have also been described in SARS-CoV-2, as well as cell-mediated immunity in GBS. Antibody-mediated mechanisms seem to be of less importance in the pathogenesis of this viral disease entity [46]. A negative SARS-CoV-2 RT-PCR test in CSF usually suggests against direct viral entry into the CNS; however, false-negatives might occur in early stages of the disease course [47]. The detection of human coronavirus (CoV) in patients’ brains can indicate that the brain may be a long-period viral reservoir without causing neurological symptoms [48]. Moreover, a post-mortem case series did not find an association between the presence of SARS-CoV-2 in the CNS and the severity of neuropathological changes [49]. The penetration of the coronavirus into the nervous system may be related to the spread from peripheral tissues through peripheral nerves to the CNS. The angiotensin converting enzyme 2 (ACE-2) receptor may also have a role in the spread of the virus, because SARS-CoV-2 binds to its enzymatic domain. ACE-2 receptors are exposed on the surface of several cell types (e.g., endothelial, epithelial, but also neuroepithelial and neurons) which may facilitate the entry of the virus into the nervous system. Dysfunction of the olfactory system in the form of hyposmia or anosmia, as the most common symptoms of SARS-CoV-2 infection, is evidence of damage to the cranial nerves. Cranial neuropathies in COVID-19 also appear as ageusia, ocular motor palsies, and trigeminal function impairment [50,51,52]. Post-infectious symptoms, such as the sensation of a blocked nose or of burning result from the affection of nasal chemesthesis and are mediated via the trigeminal nerve. These findings may suggest a potential route of penetration of SARS-CoV-2 through the intranasal trigeminal nerve endings. Spread of the virus from nasal epithelial cells to the olfactory bulb has also been suggested [53,54]. There are reports of the suppression of the olfactory system by the massive calcitonin gene-related peptide (CGRP) release from the overactive trigeminal afferent system. In this context, acquired anosmia may be due to functional connections between the olfactory and trigeminal system, and emerging headaches are associated with vigorous activation of trigeminal afferents [55,56].

Probable aetiology of Bell’s palsy is also associated with viral and autoimmune diseases; however, congenital conditions, traumas and idiopathy are also mentioned. Bell’s palsy incidence is approximately 15–30 cases per 100,000 people annually [57] and it affects women and men equally. Among viruses known to have a neurotrophic capacity for peripheral nerves are the following: herpes simplex virus 1 and 2 (HSV-1, HSV-2) and varicella zoster virus (VZV). Thanks to mucocutaneous exposure, they are able to enter the body and remain there in a latent form in multiple ganglia (such as autonomic, cranial or dorsal root) throughout the neuroaxis. Under favorable conditions, they can become reactive in an immunocompetent host. Perhaps the neural dysfunction related to HSV-1 is inducted by the activation of intra-axonal degradation and apoptotic pathways (direct and indirect responses) or, similar to GBS, by a cell-mediated immune response against myelin [58]. Mechanisms leading to the appearance of idiopathic Bell’s palsy include the following: inflammatory induced demyelination, ischemia of vasa nervorum and vascular damage. The latter are mentioned among the probable causes of facial nerve ischemia in COVID-19; however, an inflammatory process cannot be ruled out either [59]. Isolated unilateral Bell’s palsy in the context of atypical COVID-19 presentation was reported in the literature [60,61] with a delay of about 7–10 days between infection and the onset of neurological symptoms. In the cross-sectional study carried out on 41 patients with acute peripheral facial paralysis as the only symptom, almost one fourth obtained a positive SARS-CoV-2 IgG + IgM test [62].

Bilateral facial weakness is one of the prognostic risk factors contributing to mechanical ventilation in GBS [63]. The mortality rate of GBS varied between studies from 2% to even 20% [64]. Mechanical ventilation is needed in more than one fifth of patients due to respiratory failure. Unfortunately, up to 60% of intubated patients develop major complications in the form of sepsis, pneumonia or pulmonary embolism [65]. Extended follow-up of 12 months after hospitalization reported a post-GBS mortality rate of 3.9% [66]. COVID-19 infection may also worsen the outcomes of GBS patients. This co-infection contributed to both frequent arterial hypotension and admission to the ICU and worse patient Medical Research Council (MRC) sum scores [67].

Authors are debating increasingly the possible effects of COVID-19 on the onset of peripheral facial nerve palsy. Opinions are divided: Özdemir Ö. [68] in his letter to editors rejects the possibility of coincidental Bell’s palsy alongside SARS-CoV-2 infection in the pediatric population. Other studies report that the connection between COVID-19 infection and peripheral facial nerve palsy should be confirmed by further laboratory and post-mortem studies, since the number of patients with Bell’s palsy during the pandemic seems to be at the same level compared to previous years [69]; however, the available data differ [70,71]. At the same time, Egilmez O. et al. support the hypothesis that the SARS-CoV-2 virus can influence the appearance of Bell’s palsy symptoms through increasing hypercoagulopathy or direct toxic effect to the nerve [72]. It is also worth emphasizing that the suspicion of SARS-CoV-2 infection 14 days prior to the appearance of facial nerve palsy may be deceptive due to the multiplicity of clinical symptoms of viral infection [73]. 

Recently, Nasuelli et al. reported a patient who developed four-limb distal paraesthesia, bilateral facial diplegia and postural instability ten days after vaccination with Oxford–AstraZeneca chimpanzee adenovirus vectored vaccine ChAdOx1 nCoV-19 [74]. Perhaps the SARS-CoV-2 antigen(s) or chimpanzee adenovirus adjuvant contained in the vaccination may induce immune mechanisms leading to myelitis [75] or the vaccine-associated autoimmunity of DNA vaccines (ChAdOx1 and Ad26.COV2⋅S) is connected with cross-reaction between antibodies against the spike protein and peripheral nerve constituents [76]. This pathophysiology still remains unknown and requires further research. For the sake of public health, the clear information on the emergence of post-vaccination complications remains of paramount importance; however, it still remains limited. Available reports suggest a potential small but statistically significant safety concern for GBS following receipt of the Ad26.COV2.S vaccine (Janssen/Johnson & Johnson). Despite the study limitations, the absolute risk of GBS following this vaccination seems to be extremely small and much lower than the risk of COVID-19 infection [77]. The research conducted in Mexico [78] showed that GBS is infrequent among recipients of the BNT162b2 vaccine (Pfizer-BioNTech). The most cases of GBS emergence after vaccination were related to concomitant factors identified as gastrointestinal infections. This fact may indicate a lack of mechanistic connection between mRNA vaccines and GBS and vaccine safety. Also, in the case-control study, no association was found between the acute facial nerve palsy and recent vaccination with the BNT162b2 vaccine. Unlike the extremely rare reports of facial nerve palsy after vaccination, the administrations for facial nerve palsy seem to remain at a similar level compared to previous years [79]. 

In order to avoid complications of potential adverse events of COVID-19 vaccination, medical professionals should be aware of symptoms such as peripheral nerve palsy or suggestive of GBS [80]. Nevertheless, the vaccines’ benefits appear to outweigh the potential risks of developing either of these complications.

The presence of uncommon characteristics or confounding factors (e.g., pure dysautonomic presentation of GBS [81,82]) can lead to a misdiagnosis, serious health consequences, and even death [83]. For this reason, clinicians should be alerted to the wide spectrum of symptoms occurring in GBS or COVID-19 infection.

This systemic review has several limitations. The included studies are mostly clinical reports and focus on specific neurological symptoms. The number of reports is limited and the studies are retrospective. Due to the ongoing pandemic, many related studies have not yet been published, which could influence the results. There is also a high risk of cross-infection, which limited the evidence of neurological involvement.

## 6. Conclusions

In patients with a confirmed COVID-19 infection, bilateral facial palsy may be an isolated symptom as well as a variant of Guillain-Barré Syndrome. Except for one patient, the presented cases had favorable outcomes ranging from slight to almost complete recovery from facial palsy. Olfactory and gustatory dysfunctions, as well as the appearance of oculomotor palsies and trigeminal impairment in the course of this viral infection, are evidence of the involvement of the cranial nerves, which would explain also the observed facial nerve damage. 

GBS is reported as one of the more common neurological complications of SARS-CoV-2, especially since the connection between this disease entity and viral infections has already been proven. It seems reasonable to recognize and include human coronavirus as another GBS potential trigger. 

Medical professionals should be aware of potential adverse events of COVID-19 vaccinations, among which Bell’s palsy and symptoms suggestive of GBS may appear. Nevertheless, the vaccines’ benefits appear to outweigh the potential risks of developing either of these complications.

The current epidemiological situation and extremely frequent neurological complications related to the COVID-19 infection pushed the researchers to a wide analysis of its pathogenic mechanisms and manifestations. The etiopathogenesis is still unknown and requires further research. It appears that, due to negative PCR SARS-CoV-2 results in CSF, immune/cell-mediated reaction against SARS-CoV-2 should be considered rather than direct viral origin pathology; however, a potential route of penetration through the intranasal trigeminal nerve endings is also suggested. In order to clarify the spectrum of neurological manifestations and a causal relationship between SARS-CoV-2, COVID-19 vaccination and neurological symptoms, direct attention towards the study of this virus is crucial.

## Figures and Tables

**Table 1:** Summary of clinical findings and diagnostic investigations of bilateral facial nerve palsy combined with SARS-CoV-2 infection.

No.	Article	Sex	Age	Previous COVID-19 Symptoms	Time between Events	Symptoms during Admission	CSF	MRI	Neurophysiological Studies	Treatment and Outcome
1.	A. Cabrera-Muras et al. [8]	M	20	odynophagia, fever, asthenia	2 weeks	-bilateral facial paresis -positive NPS RT-PCR SARS-CoV-2 test result -Positive Epstein–Barr virus infection test	-ACD, -Negative results for SARS-CoV-2 and other viruses -Negative antiganglioside antibodies (IgM and IgG) in serum and CSF	Brain: diagnosis of bilateral facial neuritis	a severe neuropathy of the facial nerve bilaterally, with active denervation	-corticosteroids, -almost complete facial palsy recovery after 3 weeks
2.	M. Khaja et al. [9]	M	44	Asymptomatic	No data	-Bilateral complete lower motor neuron facial weakness, -Positive NPS RT-PCR SARS-CoV-2 test result	-ACD, -Negative results for SARS-CoV-2 and other viruses	Brain: N	No data	-IVIG, -slow improvement
3.	A. Sancho-Saldaña et al. [10]	F	56	fever, dry cough and shortness of breath, positive NPS RT-PCR SARS-CoV-2 test result	2 days	-low back pain -progressive proximal limb weakness with global areflexia, -bilateral facial nerve palsy, oropharyngeal	-ACD, -Negative results for SARS-CoV-2	Spine: brainstem and cervical leptomeningeal enhancement	Demyelinating neuropathy	-IVIG, -Gradual improvement
4.	J. Kerstens et al. [11]	M	27	Asymptomatic positive NPS RT-PCR SARS-CoV-2 test result	5 weeks	-asymmetrical bilateral peripheral facial palsy	Normal	Brain: bilateral contrast enhancement of the facial nerves	No evidence for GBS.	-Corticosteroids, -antiviral medication, -almost complete recovery two months later
5.	G. Toscano et al. [12]	M	23	Fever, sore throat	10 days	-complete facial palsy, -generalized areflexia, -sensory ataxia -positive NPS RT-PCR SARS-CoV-2 test result	-ACD, -Negative results for SARS-CoV-2	Brain: bilateral contrast enhancement of the facial nerves Spine: N	Axonal sensory-motor damage	-IVIG, -mild improvement
6.*	P. Jain et al., Atypical Presentation of Guillain-Barré Syndrome (GBS) with Facial Diplegia and Retained Reflexes associated with COVID-19 Infection	M	43	flu-like illness with fever, headache and generalized body pain, suspicion of mild COVID-19 infection	3 weeks	-bilateral peripheral facial nerve palsy	-ACD, -Negative results for SARS-CoV-2 and other viruses	Brain: subtle enhancement of seventh cranial nerve	No data	-IVIG, -Rapid improvement
7.*	V. Pandya et al., COVID-19 Associated Facial Diplegia and Lower Extremities Weakness; Subtype of GBS: A Case Report	F	48	Diagnosed COVID-19	3 weeks	-bilateral facial muscles weakness, -symmetrical and proximal lower extremities weakness, -bilateral loss of deep tendon reflexes in the lower extremities	-ACD, -negative PCR for common viral and bacterial pathogens	No data	No data	-IVIG, -Significant improvement
8.	C. Judge et al. [13]	M	64	cough, fever and chills, positive RT-PCR SARS-CoV-2 test result	3 weeks	-Peripheral bilateral facial nerve palsy, more pronounced on the right	-ACD with lymphocytic pleocytosis -Tests for different viruses were negative	Brain: N	No data	-No data, -Gradual improvement
9.	J. Caamaño et al. [14]	M	61	fever and coughing, positive NPS RT-PCR SARS-CoV-2 test result	10 days	-bilateral facial nerve palsy -unresponsive blink reflex on both eyes	-ACD, -negative RT-PCR for SARS-CoV-2	Brain: N	No data	-Corticosteroids, -barely notable improvement
10.	N. Mackenzie et al. [15]	F	39	ageusia, anosmia and intense headache	14 days	-peripheral facial diplegia, -generalized quadriparesis and hyporeflexia, -positive NPS RT-PCR SARS-CoV-2 test result	ACD	Spine: result not related with the clinical signs and symptoms	confirmed the GBS diagnosis	-Plasmapheresis, -Corticosteroids, -Gradual improvement
11.	T. Pelea et al. [16]	F	56	dry cough, mild fever and a general weakness, positive NPS RT-PCR SARS-CoV-2 test result	7 days	-gradual progression, paresis in four limbs, -absent deep tendon reflexes, -sensory disturbances -5 days later: severe tetraparesis and bilateral peripheral facial nerve palsy	-ACD, -negative RT-PCR for SARS-CoV-2	Spine: N	axonal demyelinating neuropathy	-IVIG, -only slight improvement
12.	M. Abolmaali et al. [17]	M	47	Dyspnea and cough	10 days	-Dysarthria, -mild muscle weakness and generalized hyporeflexia -severe low back pain with quadriparesis -areflexia, -bilateral facial palsy	ACD	Brain and spine: N	acute motor-sensory axonal (AMSAN) neuropathy	-Corticosteroids, -died from severe autonomic dysfunction
13.	J.L. Chan et al. [18]	M	58	Exposed to SARS-CoV-2 in the workplace, asymptomatic	20 days	-complete facial diplegia and areflexia in the lower extremities, -dysarthria -positive NPS RT-PCR SARS-CoV-2 test results	ACD, negative RT-PCR for SARS-CoV-2	Brain: bilateral facial nerve enhancement	acute inflammatory demyelinating polyneuropathy	-IVIG, -slight improvement
14.	J. Aasfara et al. [19]	F	36	Diagnosed COVID-19	6 weeks	-reduced tendon reflexes, -left peripheral facial palsy -After 24 h, right peripheral facial palsy and asymmetric distal numbness -Blood serology revealed IgG SARS-CoV-2 antibody	ACD, PCR for several viruses, including, SARS-CoV-2, were negative	Brain and spine: N	demyelinating pattern of GBS	-IVIG, -Gradual improvement

F–female, M–male, CSF–cerebrospinal fluid, GBS–Guillain–Barré syndrome, ACD–albumin-cytological dissociation, IVIG–intravenous immunoglobulin, MRC–Medical Research Council, RT-PCR–reverse transcription polymerase chain reaction, N–normal, NPS–nasopharyngeal swab, MRI–Magnetic resonance imaging * -only abstract (https://n.neurology.org/content/96/15_Supplement/4182, https://n.neurology.org/content/96/15_Supplement/4246) (accessed on 30 June 2021).
